# MiR‐3130‐5p is an intermediate modulator of 2q33 and influences the invasiveness of lung adenocarcinoma by targeting NDUFS1

**DOI:** 10.1002/cam4.3885

**Published:** 2021-05-12

**Authors:** Juan Zhan, Shenghua Sun, Yixing Chen, Chaoqun Xu, Qinwei Chen, Minjie Li, Yihua Pei, Qiyuan Li

**Affiliations:** ^1^ Department of Pulmonary and Critical Care Medicine The Third Xiangya Hospital Central South University Changsha China; ^2^ Department of Oncology Zhongshan Hospital Xiamen University Xiamen China; ^3^ Laboratory Xiamen Cancer Center The First Affiliated Hospital of Xiamen University Xiamen China; ^4^ National Institute for Data Science in Health and Medicine School of Medicine Xiamen University Xiamen China; ^5^ Department of Thoracic Surgery Zhongshan Hospital Xiamen University Xiamen China; ^6^ Central Laboratory Zhongshan Hospital Xiamen University Xiamen China

**Keywords:** 2q33, expression quantitative trait loci, lung adenocarcinoma risk loci, miR‐3130‐5p, NDUFS1

## Abstract

Genome‐wide association studies (GWAS) have reported a handful of loci associated with lung cancer risk, of which the pathogenic pathways are largely unknown. We performed *cis*‐expression quantitative trait loci (eQTL) mapping for 376 lung cancer related GWAS loci in 227 TCGA lung adenocarcinoma (LUAD) and reported two risk loci as eQTL of miRNA. Among the miRNAs in association with lung cancer risk, we further predicted and validated miR‐3130‐5p as an intermediate modulator of risk loci 2q33 and the tumor suppressor NDUFS1. We assessed the phenotypic impacts of the interaction between miR‐3130‐5p and NDUFS1 in both lung cancer cell lines and mice xenograft models. As a result, miR‐3130‐5p directly regulates the expression of NDUFS1 and the corresponding tumor invasiveness, migration and epithelial‐mesenchymal transition (EMT). Our findings provide important clues for the pathogenic mechanism of 2q33 in lung carcinogenesis which informs clinical diagnosis and prognosis of LUAD.

We performed a *cis*‐eQTL analysis for 376 lung cancer risk loci based on the expression profiles of 251 miRNAs in a cohort of 227 TCGA lung adenocarcinoma. We report a novel pathogenic pathway of 2q33 via miR‐3130‐5p and NDUFS1.

## Graphical abstract

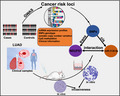

## 1 INTRODUCTION

Genetic variations cause multiple human complex diseases, including cancers. GWAS have identified a vast amount of SNPs associated with diverse human traits by comparing large cohort of cases and healthy controls.^1^, ^2^ Previous GWAS reported approximate 680 risk loci for lung cancer and lung‐related diseases. However, most of the trait‐associated loci (TAL) are located in the non‐coding genomic regions^3^ and do not change the amino acid sequences of the protein, suggesting that these GWAS risk SNPs act as regulatory elements via influencing gene expression. Therefore, understanding the expression regulatory activities of TAL in non‐coding regions becomes a demanding question of medical genetics and a milestone of the clinical implications.

eQTL serves as a bridge between non‐coding germline variants and variations of phenotypes, and thereby reveals the molecular mechanisms of TAL.^4^ The rationale underlies eQTL is that germline variations alter the transcription abundances of mRNA or other transcripts to cause phenotypic changes in the cell. Many studies demonstrated that eQTL can further affect a wider spectrum of transcripts by targeting at genes with *trans*‐acting regulatory activities.^5^, ^6^ Several large data consortia have been established by eQTL mapping based on large parallel sequencing projects. For example, the “Genotype‐Tissue Expression” (GTEx) Project identified eQTLs in normal human tissues; and Gong et al. established “PancanQTL,” “ncRNA‐eQTL,” and “GPSno” databases for *cis*‐ and *trans*‐eQTLs of non‐coding RNAs, protein coding RNAs and small nucleolar RNAs respectively in 33 cancer types from TCGA.^7^, ^8^, ^9^, ^10^ In particular, many recent studies successfully elucidated the susceptibility pathways of cancer risk loci by eQTL analyses in cancer cohorts, such as 15q25.1 and 8q24.^11^, ^12^, ^13^

The landscape of eQTLs is highly specific to cell lineage, differentiation statuses as well as tissue types.^14^ Therefore, eQTL mapping for cancer expression is highly informative of cancer biology. Several prior studies identified the regulatory axes for eQTLs in cancers and thereby revealed the biological processes that drive carcinogenesis. The discovery of eQTLs in cancers and the corresponding regulatory axes can inform cancer studies in two ways: first, eQTLs can be used to elucidate the functions of the non‐coding cancer risk loci and thereby fine‐map the causal pathogenic alleles; second, the effectors of the cancer eQTL are highly potential oncogenes or tumor‐suppressors and serve as biomarkers in cancer diagnosis and therapy.

MiRNA is a class of endogenous small non‐coding RNAs which functions as transcription regulator to inhibit gene expression by directly binding to the miRNA response element (MRE) in the 3'UTR of target genes. Accurate computational identification of miRNA targets is particularly important to investigate biological mechanisms. Many software and web servers have been developed to predict miRNA targets mainly based on evolutionary conservation of binding sites.^15^, ^16^, ^17^, ^18^ Dysregulation of miRNAs has been associated with diverse diseases, including lung cancer.^19^ Several miRNAs have been used as novel biomarkers and therapeutic targets of cancers.^20^, ^21^

Here in our study, we performed a *cis*‐eQTL mapping for a set of lung‐related TAL from GWAS and reported an association between 2q33 and a cancer‐associated miRNA, miR‐3130‐5p. We further investigated the function impacts of the association in lung adenocarcinoma (LUAD). As a result, we reported NDUFS1 as a target gene of 2q33‐miR‐3130‐5p association in LUAD and validated the corresponding phenotypic impacts on invasion, migration and EMT based on in vitro and in vivo analyses.

## 2 MATERIALS AND METHODS

### 2.1 Data and materials

We obtained 376 risk loci associated with traits of lung cancer from the GWAS catalog on 10 October 2017 (Table S1).^22^ Then we retrieved the genotype data from 490 TCGA lung adenocarcinoma. To control the population structure, we only included Caucasian descendants. We inferred the ancestry of the cohort using 2504 individuals from 1000 Genomes project with known ancestry as a reference and followed the method described in previous study.^5^

For each locus, we imputed the genotypes for variants within 1 Mb range of the tag SNP and with linkage disequilibrium (*R* ^2^ > 0.5) to the 1 KG variants with 1000 Genomes Phase 3^23^ using IMPUTE2 software.^24^, ^25^ In order to control for statistical power, we filtered the imputed variants according to the following criteria: (a) info score >0.7; (b) minor allele frequency (MAF) >5%; (c) posterior probability >0.7; (d) Hardy‐Weinberg Equilibrium test *p* value >1 × 10^−6^.

We obtained the miRNA expression profiles of 227 TCGA‐LUAD, with matched somatic copy number alteration (SCNA) data and CpG methylation data.^26^ The miRNA expression levels were transformed based on log_2_(RPM+1). To control the effect of outliers, we excluded individuals of which the miRNA expression levels were greater than three times of the standard deviation. Then we removed miRNA which were absent in over 20 individuals. For the simplicity of the computation, the copy number variation of a given miRNA the SCNA was categorized into gain (+1), neutral (0), loss (−1). For the CpG methylation measure, we used categorized levels of 0–3 based on the beta values of probes located within 200 and 1500 bp of the transcription start sites (TSS200 and TSS1500).

### 2.2 *Cis*‐eQTL analysis for TAL in relation with LUAD

We performed *cis*‐eQTL mapping for 3291 SNPs in LUAD using R package, “MatrixEQTL”.^27^ For each SNP, we assessed the associations between the genotypes and the expression levels of miRNA located within 1 Mb either side of the SNP. To control confounding effects, we adjusted the expression levels of miRNA using somatic copy number variation, CpG methylation, sex and age of patients via the following multivariate regression model:

(1) $$miRNA_{i}=\beta_{0}+\beta_{1}G_{i}+\beta_{2}Age_{i}+\beta_{3}Sex_{i}+\beta_{4}CNV_{i}+\beta_{5}CpG_{i}+\varepsilon_{i}$$

Here, $\varepsilon_{i}∼N\left(0,\sigma^{2}\right)$ is a Gaussian error term; $G_{i}$ is the genotype of the ith sample; ${\mathrm{Age}}_{i}$ is the age of the ith sample; ${\mathrm{Sex}}_{i}$ is the sex of the ithsample; ${\mathrm{CNV}}_{i}$is the copy number variation of the ith sample; ${\mathrm{CpG}}_{i}$ is the methylation status of the ith sample; $\beta_{0}$ is the intercept, other β are the regression coefficients. We called significant *cis*‐associations between eSNP and the target miRNA (eMiR) based on a false‐discovery rate (FDR) of 0.1.

To identify differently expressed miRNA in LUAD, the expression levels of miRNAs which were absent in more than 10 samples in normal or tumor samples were excluded. The rest were compared between tumors and normal lungs using *t* test. MiRNAs with fold change >2 and FDR <0.01 were defined as differently expressed miRNAs.

### 2.3 Target gene prediction

In order to predict the downstream target mRNAs of eMiR, we used miRNA Data Integration Portal (mirDIP 4.1)^18^ to predict target mRNAs with corresponding miRNA binding motifs in the 3'UTR regions. Then we confirmed the endogenous effect of the eSNP on the mRNA using Mendelian randomization (MR) with the eSNP as instrumental variable (Equation 2). We selected significant candidate mRNAs based on the following criteria: (a) Target genes (“Mir‐target gene”) Integrated Score ≥0.1; (b) filtering these genes (“Present calls”) with no less than 100 present calls in the cohort; (c) genes to which the TAL is significant genetic instrument based on Mendelian Randomization (“MR gene”, weak instrument test FDR <0.05); (d) Target genes are required to be significantly differently expressed (“DE gene”) between tumor tissue and the matched normal (FDR<0.01 and log_2_fold change>0.5); (e) significantly predictive (“Predictive gene”) of the overall survival rate using KM‐Plotter software.^28^

(2) $$mRNA_{i}=\beta_{0}+\beta_{1}miRNA_{i}|G_{i}+\varepsilon_{i}$$

Here, $\varepsilon_{i}∼N\left(0,\sigma^{2}\right)$ is a Gaussian error term; *G_i_* is the genotype of the *i*th sample;$\beta_{0}$ is the intercept,$miRNA_{i}$ is miRNA expression level of the *i*th sample, $\beta_{1}$ is the regression coefficients.

### 2.4 Cell lines and cell culture

Human LUAD cell lines (A549, H1299, H1395, H1975, Calu‐3, and H1650) were obtained from the Cell Bank of Chinese Academy of Sciences (Shanghai, China). A549 was maintained in F‐12 K Medium (Gibco, USA). H1299, H1395, H1975, Calu‐3, and H1650 were maintained in RPMI‐1640 Medium (Gibco, USA). All cells were maintained in medium with 10% FBS (Gibco, USA), 1% penicillin/streptomycin (Gibco, USA) in a humidified environment containing 5% CO_2_ at 37°C. The bio‐banking procedures were strictly followed and cells were used within 3 months.

### 2.5 Cell transfection

A549 and H1650 cells were transfected with miR‐3130‐5p agomir (sense:5′‐ACCCAGUCUCCGGUGCAGCCU‐3′; antisense:5′‐AGGCUGCACCGGAGACUGGGU‐3′, Sangon Biotech, China), miR‐3130‐5p agomir control (sense:5′‐UUCUCCGAACGUGUCACGUTT‐3′; antisense:5′‐ACGUGACACGUUCGGAGAATT‐3′), miR‐3130‐5p antagomir (5′‐AGGCUGCACCGGAGACUGGGU‐3′) and miR‐3130‐5p antagomir control (5′‐CAGUACUUUUGUGUAGUACAA‐3′). The overexpression and knockdown plasmids of NDUFS1 were purchased from Genechem, China. Quantitative reverse transcription–polymerase chain reaction (qRT‐PCR) and western blotting were conducted to detect the transfection efficiency. For rescue experiment, we treated with miR‐3130‐5p agomir after stable NDUFS1 overexpression in cell lines.

### 2.6 Dual‐luciferase reporter assays

The 3′‐UTR fragments of NDUFS1 and RGS11 predicted to interact with miR‐3130‐5p were amplified and cloned into pmirGLO Dual‐Luciferase miRNA Target Expression Vector. A549 cells transduced by those plasmids were seeded in a 24‐well plate and transfected with miR‐3130‐5p agomir, miR‐3130‐5p antagomir and controls for 48 h. The cells were lysed and the luciferase activities of firefly and renilla were examined with Dual‐Luciferase Reporter assay kit following the manufacturer's instructions. All experiments were performed three times.

### 2.7 Patients and tissue samples

Primary LUAD tissues and paracancerous tissues were obtained from 43 patients who received lung cancer resections at the Department of Thoracic Surgery of Zhongshan Hospital affiliated to Xiamen University. Written informed consents were acquired by all patients participated in the study. Our protocols were approved by Medical Ethic Council for Researchers of Zhongshan hospital and executed according to the Declaration of Helsinki. The diagnoses of LUAD were made by two independent pathologists. The resected tissue samples were quickly frozen in liquid nitrogen and stored at −80°C.

### 2.8 RNA extraction and qRT‐PCR

Total RNAs were extracted from cells and human tissue samples using TRIzol reagent (Invitrogen, Carlsbad, CA, USA) and purified using the PureLink RNA Mini kit (Invitrogen) according to the manufacturer's protocols. Approximately 3.5 µg of RNA was reverse‐transcribed to DNA templates for qRT‐PCR using the Reverse‐Aid™ First Strand cDNA Synthesis kit (Fermentas, Waltham, MA, USA).

### 2.9 Cell viability assay

Cells (4 × 10^3^) were placed in 96‐well plates and assessed every 24 h. CCK‐8 reagent (Sangon biotech, China) was added and incubated with cells for 1 h at 37°C before the absorbance at 450 nm was measured by a microplate reader.

### 2.10 Transwell assay

Cells (5 × 10^4^) suspended in 200‐μl medium without serum were seeded into the upper chamber, and lower chamber was full of 20% FBS to induce LUAD cells to migrate through the membrane. Matrigel (1:6 dilution, BD Biosciences, USA) was added on the upper chamber for the invasion assay. Twenty‐four hours later, cells were fixed in 4% paraformaldehyde, stained with 0.1% crystal violet solution and counted under optical microscope.

### 2.11 Wound‐healing assay

Cells (2 × 10^6^) were seeded and incubated in fresh six‐well plates. For the wound‐healing assay, a confluent monolayer was scratched with a pipette tip, washed with PBS and incubated in culture medium supplemented with 1% FBS. The cultures were imaged by microscope after 0 and 48 h.

### 2.12 Colony formation assay

Cells (200) were seeded and incubated in fresh 6‐well plates for 14 days to allow colony formation. Colonies were fixed in 4% paraformaldehyde, stained with 0.1% crystal violet solution and counted.

### 2.13 TUNEL staining

Cells were stained using TUNEL apoptosis detection kit (DeadEnd Fluorometric TUNEL system, Promega, USA) according to manufacturer's instructions. Nuclei were stained with 4′,6‐diamidino‐2‐phenylindole (DAPI). The results were detected using a Leica DMI3000 immunofluorescent microscope.

### 2.14 Protein extraction and western blotting

Cells or tissue samples were lysed in ice‐cold RIPA lysis buffer containing protease inhibitor. An equal amount of total protein from each sample (20 µg) was loaded on 10% SDS‐PAGE gels and then transferred to PVDF membranes. The membranes were blocked with 5% BSA for 2 h at room temperature and then incubated with commercially available primary antibodies for detection of NDUFS1 (Cell Signaling Technology, USA), followed by the appropriate secondary antibodies (Cell Signaling Technology, USA). GAPDH was taken as internal control and protein bands were analyzed by Image J software (NIH, USA).

### 2.15 Mice xenograft experiment

A549 cells (2.5 × 10^6^) were injected subcutaneously into the flanks of Balb/c nude mice aged 5 weeks. After formation of tumor (4 ×4 mm), mice were treated with either control or miR‐3130‐5p agomir (2 nmol twice a week) via tail vein for totally 4 weeks. The mice's condition, body weights and sizes of tumors were observed and recorded every three days. The tumor volumes were recorded in (width^2^ × length)/2. After 2 weeks from last treatment, the animals were euthanized and tumors were removed, weighed, and frozen or fixed for biochemical or histological analyses.

### 2.16 Immunohistochemistry

The paraffin‐embedded tissue slides (4 μM) were deparaffinized and hydrated. Tumor sections were immunostained subsequently for NDUFS1, CD34, Collagen‐IV. MaxVisionTM kit was applied to each slide (MaixinBiol, China) according to the manufacturer's instructions. Color was developed by DAB kit (MaixinBiol, China) and slides were counterstained with hematoxylin. Nonimmune mouse IgG was used to replace primary antibodies for negative controls. The immunostained slides were observed under microscope and scored by two independent pathologists using Image J.

### 2.17 Clinical data collection and statistical analysis

The medical records of patients enrolled in the study were reviewed and approved by Medical Ethic Council for Researchers of Zhongshan Hospital Affiliated to Xiamen University with patients’ written consents. The information of demographic characteristics, pathological characteristics of primary tumors, diagnoses and therapies were collected. These patients were followed up every 3 months until deaths or end of the investigation.

All the experiments were conducted in triplicates with consistent results. All data were listed as mean with SD and analyzed by SPSS 20.0 (IBM, USA) and Graphpad Prism 7.0 (GraphPad Software Inc.). Student's *t* test was adopted to confirm statistical significance. *p* value less than 0.05 was regarded as statistically significant. We used logistic regression to evaluate the association between NDUFS1 or miR‐3130‐5p and corresponding clinical features and performed survival analysis based on cox regression using “survival” and “survminer” R packages (version: R‐4.0.2).

## 3 RESULTS

### 3.1 *Cis*‐eQTL analysis for GWAS trait‐associated loci in TCGA LUAD Cohort

We retrieved 376 loci which were annotated to associate with disease traits in human lung, each of which is represented by a tag SNP (Table S1). Based on each tag SNP, we obtained 3291 SNPs in linkage disequilibrium (*R* ^2^ > 0.5). The missing genotypes of the SNPs were imputed from the genotyping data of TCGA cohort.

To define the study population, we stratified 490 TCGA LUAD samples based on the top two principal components of common variants (see Section 2). After filtering for samples with missing or extreme values, we obtained a cohort of 227 Caucasian descendants with LUAD.

**FIGURE 1:**
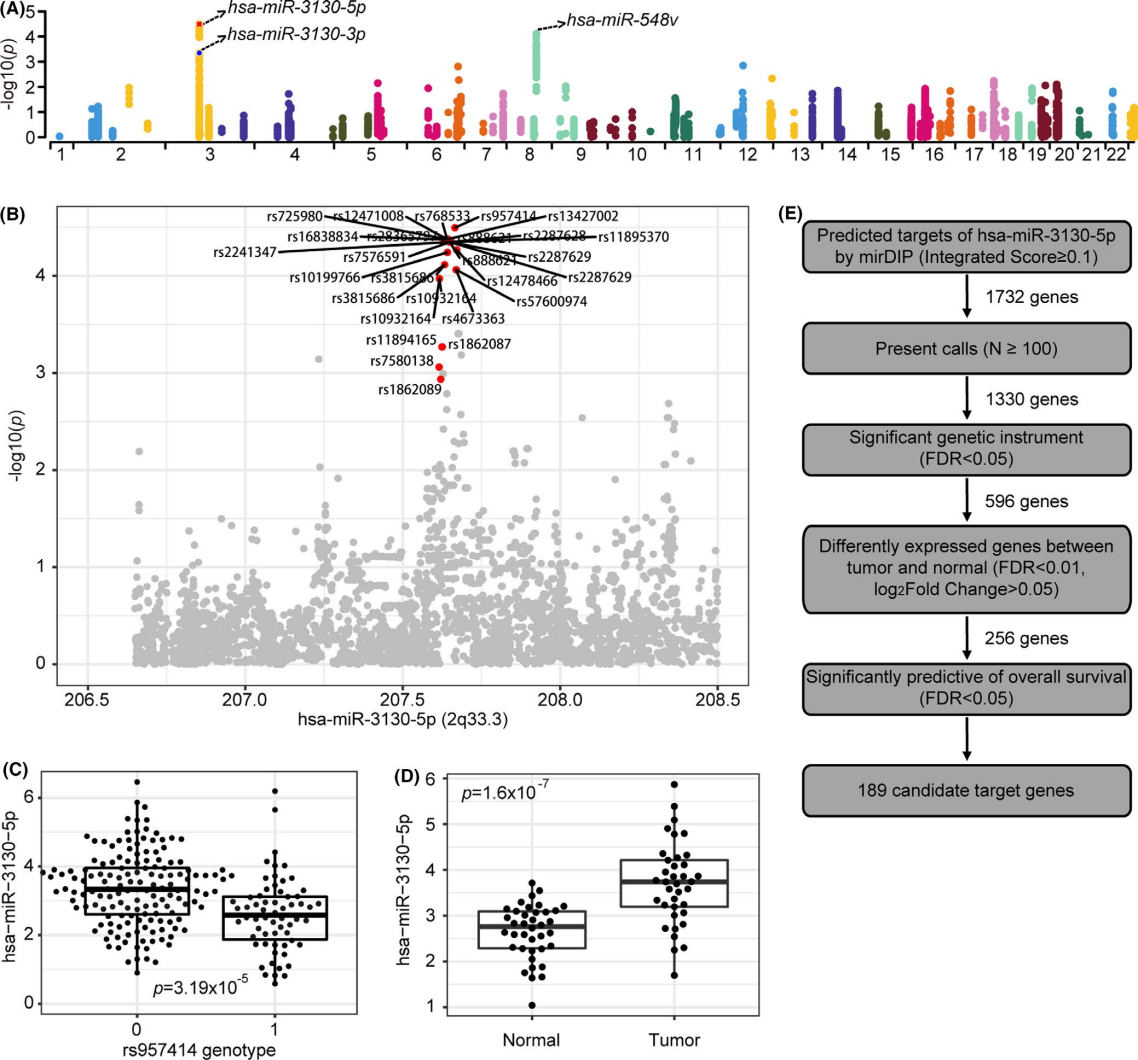
The eQTL analyses of lung cancer risk loci and prediction of candidate target genes of 2q33‐miR‐3130‐5p. (A) Manhattan plot of *cis*‐eQTLs in LUAD along the 22 human chromosomes. (B) Regional association plot of miR‐3130‐5p. (C) Association between genotype of significant rs957414 SNP and expression of miR‐3130‐5p. (D) Boxplot of expression of miR‐3130‐5p in normal lung tissue (Normal) and lung tumor tissue (Tumor) according to TCGA‐LUAD database. (E) The schematic about the screening process of targets prediction of miR‐3130‐5p (see Section 2)

We then performed a *cis*‐eQTL analysis for 3291 SNPs and 251 miRNAs located within 1 Mb region on either side of the SNPs. Based on an FDR of 0.1, we identified 100 significant *cis*‐associations which correspond to 93 eSNPs and 3 target miRNAs (eMiR) (Figure 1A–C and Table S2). The eSNPs belong to two risk loci (2q33 and 8p22) and are both associated with lung cancer.^29^, ^30^

We evaluated the expression levels of three eMiRs in LUAD and matched normal samples. As a result, miR‐3130‐5p (log_2_fold change = 1.04, FDR =8.12 × 10^−7^; Figure 1D) and miR‐548v (log_2_fold change = 0.26, FDR = 0.01; Figure S1) were significantly differently expressed. Moreover, miR‐3130‐5p expression level is significantly predictive of the overall survival rate in a cohort of 323 TCGA LUAD patients (*p* = 0.024; Figure S2A). Therefore, we focused on miR‐3130‐5p for further analysis.

To reveal the downstream impacts of the TAL which act as *cis*‐eQTL of miR‐3130‐5p, we retrieved 1732 mRNAs (Integrated Score ≥ 0.1; Table S3) predicted by mirDIP 4.1 database (Figure 1E). These genes carry the binding motif of miR‐3130‐5p in the 3′UTR. After filtering these genes for present calls in the cohort, we yielded 1330 target genes for further analysis (Figure 1E). We performed instrumental variable (IV) regression taking the eSNPs as genetic instruments, miRNA expression as independent variable and mRNA expression as dependent variable. As a result, we yielded 11,756 regulatory axes, including 23 eSNPs of miR‐3130‐5p and 596 mRNAs in *trans* (Table S4 and Figure 1E). To identify the mRNAs regulated by miR‐3130‐5p that are associated with LUAD patients’ survivals, 256 significantly differently expressed genes in lung cancer were chosen for further survival analyses (Figure 1E). As results, 189 genes significantly predictive of overall survival (FDR <0.05; Figure 1E) were selected, among which NDUFS1 is the most significantly predictive (HR: 0.46, CI 95%: 0.38–0.55, FDR = 4.25 × 10^−5^; Figure S2B and Table S5). Other candidate genes with known functional impacts in cancer include RGS11,^31^ RPS6KA2,^32^ and CYB5A.^33^ KEGG enrichment analysis suggested the 189 candidate genes overrepresent in two cancer‐related pathways (Wnt signaling pathway and Cell cycle) at a 5% FDR level (Table S6). Based on literature research,^31^, ^34^ we chose two genes, NDUFS1 and RGS11, for further analyses.

### 3.2 NDUSF1 is a target for miR‐3130‐5p

**FIGURE 2:**
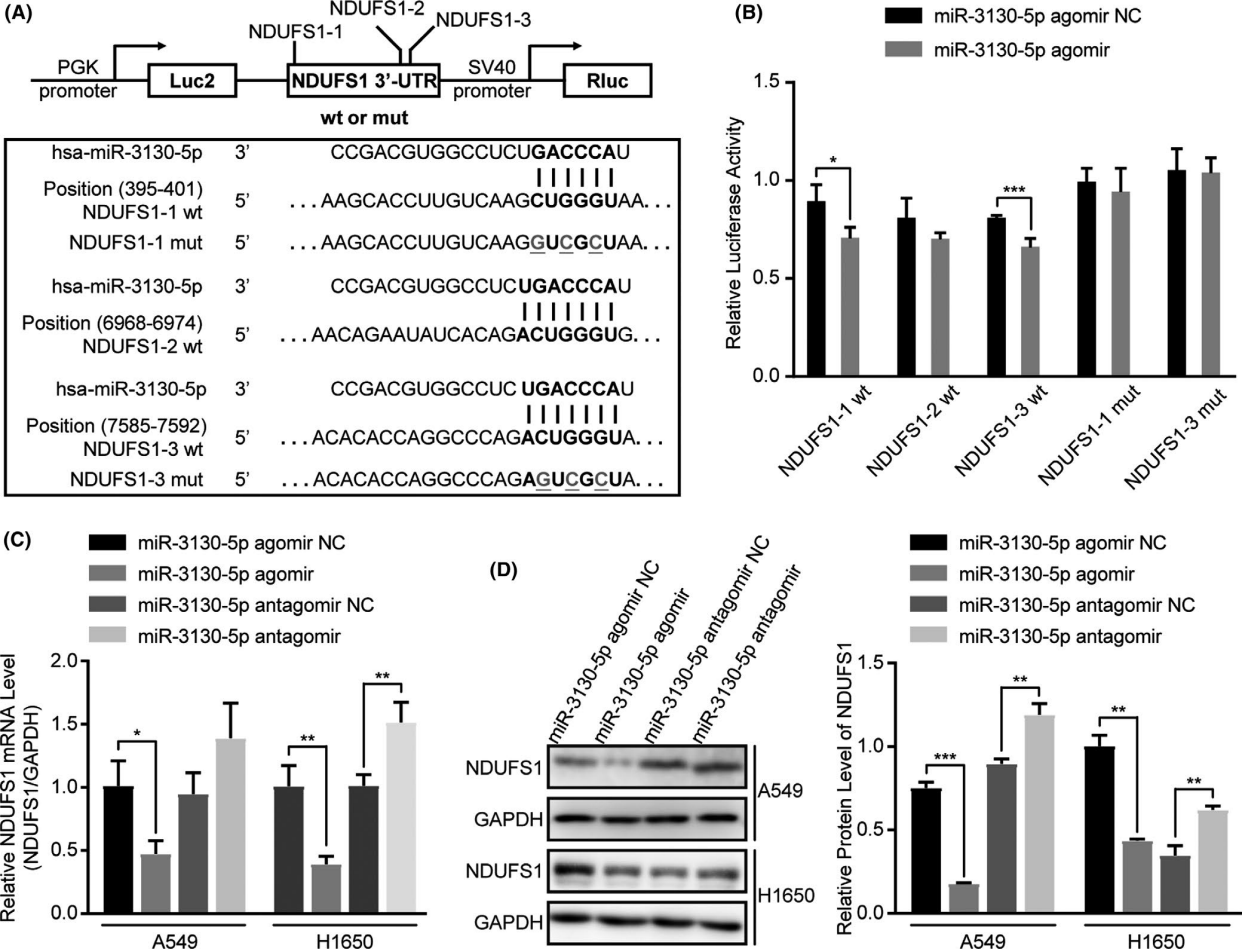
NDUFS1 is verified to be a target for miR‐3130‐5p. (A) The schematic shows wild type (wt) or mutant type (mut) NDUFS1 3’UTR (including three different predicted binding sites of miR‐3130‐5p) was cloned to the pmirGLO Dual‐Luciferase miRNA Target Expression Vector. (B) The relative luciferase activities were detected in A549 cells transfected with wild‐type or mutant‐type NDUFS1 3’UTR and miR‐3130‐5p agomir or agomir control. (C) qRT‐PCR analysis of NDUFS1 mRNA level in A549 and H1650 cells transfected with miR‐3130‐5p agomir, antagomir and respective controls. (D) NDUFS1 protein level was measured by western blotting in A549 and H1650 cells after transfection with miR‐3130‐5p agomir, antagomir and respective controls. *p < 0.05, **p < 0.01, ***p < 0.001

To verify the target gene of miR‐3130‐5p, we performed luciferase reporter assays for two predicted targets, NDUFS1 and RGS11 (Figure 2A and Figure S3B). In A549 cells, the reporter with wild‐type NDUFS1 3′UTR exhibited significantly attenuated luciferase activities in response to the overexpression of miR‐3130‐5p (agomir transfection; *p* < 0.05; Figure 2B) but showed no response to the knock‐down (antagomir transfection; *p* > 0.05; Figure S3A). In addition, the changes in the luciferase activities caused by the overexpression and knock‐down of miR‐3130‐5p were further validated by the changes of NDUFS1 mRNA levels (Figure 2C) and protein levels (Figure 2D). For RGS11, the luciferase activities were not significantly affected by miR‐3130‐5p (Figure S3C). In summary, our data suggested that NDUFS1 is the direct target mRNA of miR‐3130‐5p in LUAD.

### 3.3 miR‐3130‐5p promotes invasion, migration, and EMT in vitro

**FIGURE 3:**
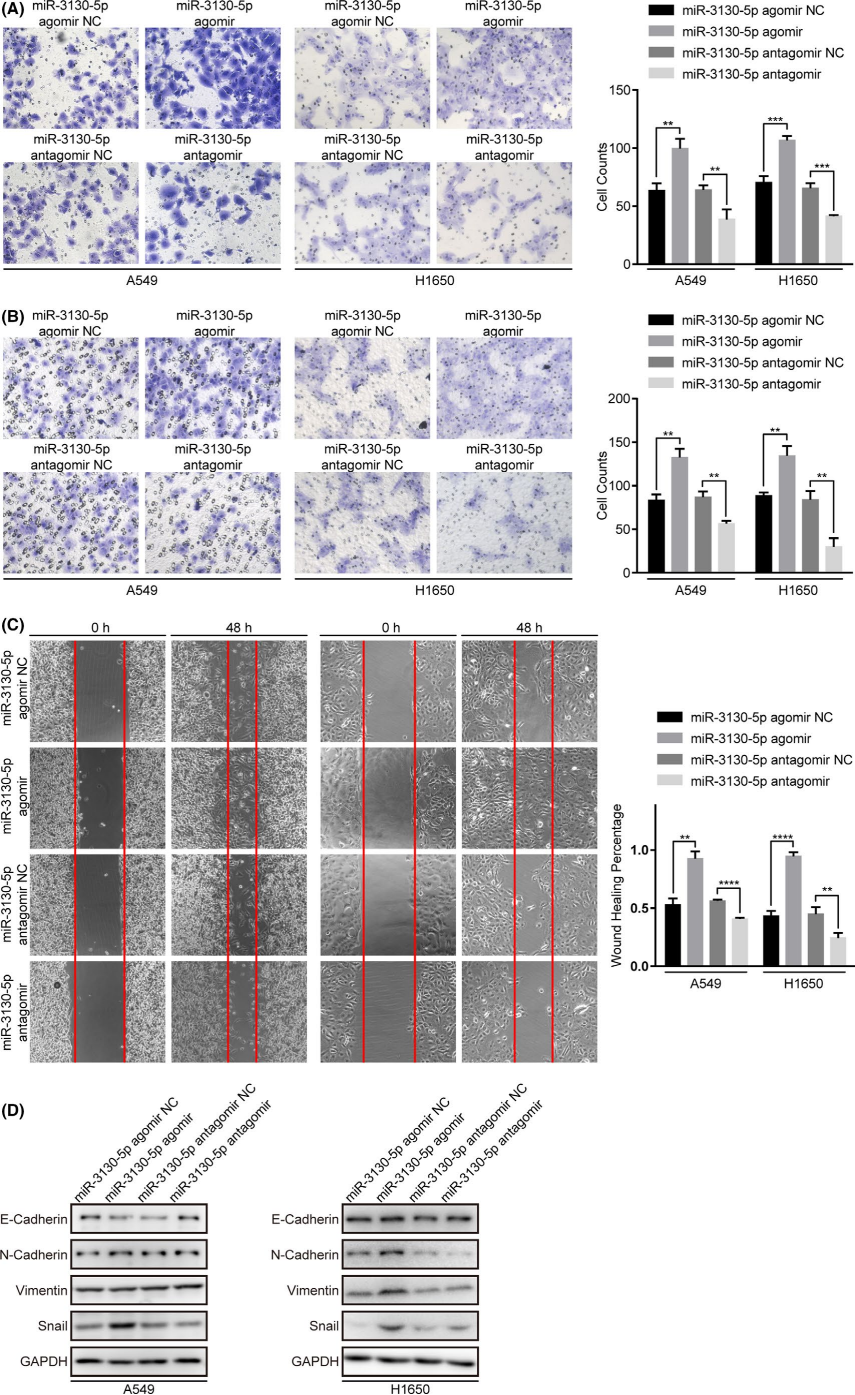
MiR‐3130‐5p significantly promotes the abilities of invasion and migration of LUAD cells. (A) Invasion abilities of A549 and H1650 cells were assessed by matrigel chamber invasion assays after treated with miR‐3130‐5p agomir, antagomir and respective controls. (B) Migration abilities of A549 and H1650 cells were assessed by transwell assays after treated with miR‐3130‐5p agomir, antagomir and respective controls. (C) Migration abilities of A549 and H1650 cells were also detected by scratch assays after 48 h of transfection with indicated substances. (D) Western blotting results of EMT related proteins levels in A549 and H1650 cells after transfected with indicated substances. All representative images are shown as above. **p < 0.01, ***p < 0.001, ****p < 0.0001

To further verify that miR‐3130‐5p functions as an oncomiRNA in LUAD, we first assessed the expression levels of miR‐3130‐5p in a panel of cell lines derived from LUAD. Our data showed miR‐3130‐5p is stably expressed in A549 and H1650, which we chose for further analysis. We then conducted a series of in vitro experiments to assess the effects of miR‐3130‐5p on the malignant phenotypes in the two cell lines. As a result, miR‐3130‐5p overexpression (agomir) significantly promoted the invasion and migration of A549 (*p* < 0.01) and H1650 (*p* < 0.001 and *p* < 0.01) cells after transfection for 24 h (Figure 3A and B). Consistently, the knock‐down of miR‐3130‐5p by antagomir substantially suppressed the two phenotypes. Additionally, the wound healing assays also ascertained that miR‐3130‐5p agomir greatly intensified the migration abilities of A549 (*p* < 0.01) and H1650 (*p* < 0.0001) cells, which were significantly reduced by miR‐3130‐5p antagomir (A549: *p* < 0.0001 and H1650: *p* < 0.01) (Figure 3C).

Furthermore, the western blotting assays showed suppressed epithelial markers (E‐Cadherin) and activated mesenchymal markers (N‐Cadherin, Vimentin, Snail) in A549 and H1650 cells driven by increased miR‐3130‐5p expression (*p* < 0.01; Figure 3D and S5), indicated miR‐3130‐5p may promote EMT by regulating the expression of Snail.

On the other hand, the CCK8 and colony formation assays suggested that miR‐3130‐5p does not affect cell proliferation and colony formation of LUAD cells (Figure S4A and B). And miR‐3130‐5p had no effect on the apoptosis process of LUAD cells according to the TUNEL assay (Figure S4C). In summary, our findings suggest that miR‐3130‐5p specifically promotes invasion, migration and EMT of LUAD cells.

### 3.4 miR‐3130‐5p modulates NDUFS1 functions in vitro

**FIGURE 4:**
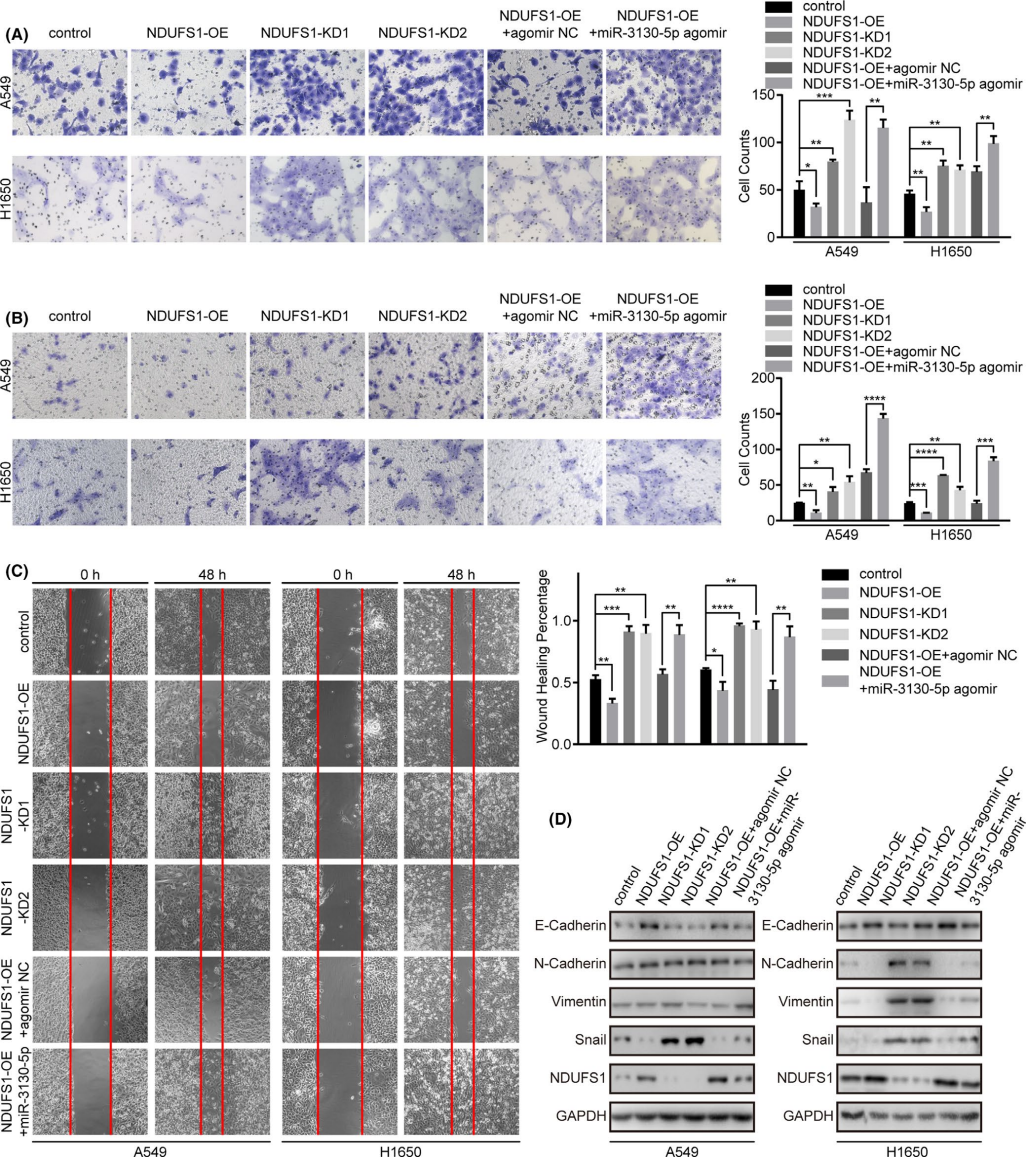
NDUFS1 significantly inhibits the abilities of invasion and migration of LUAD cells and miR‐3130‐5p can rescue partly the suppressed abilities caused by NDUFS1. (A) Invasive abilities of A549 and H1650 cells were assessed by matrigel chamber invasion assays after the above processes. (B) Migration abilities of A549 and H1650 cells were examined by the migration assays under those indicated conditions. (C) Migration abilities of A549 and H1650 cells were also evaluated by scratch assays after 48‐h transfection with different substances. (D) Western blotting results of EMT related proteins and NDUFS1 levels in A549 and H1650 cells transfected with those indicated substances. All representative images are shown as above. *p < 0.05, **p < 0.01, ***p < 0.001, ****p < 0.0001

As the target mRNA of miR‐3130‐5p, NDUFS1 showed opposite effects on invasion, migration phenotypes in both LUAD cell lines (Figure 4A and B). We assessed the phenotypic changes in response to NDUFS1 over‐expression and knock‐down in the same cell types as miR‐3130‐5p. Of note, over‐expression of NDUFS1 inhibited significantly invasion and migration in A549 (*p* < 0.05 and *p* < 0.01) and H1650 (*p* < 0.01 and *p* < 0.001) cells, while knock‐down of NDUFS1 led to a markedly elevated invasion and migration abilities of A549 (*p* < 0.01, *p* < 0.001 and *p* < 0.05, *p* < 0.01) and H1650 (*p* < 0.01, *p* < 0.01 and *p* < 0.0001, *p* < 0.01) (Figure 4A and B). Moreover, wound healing assays demonstrated that increased NDUFS1 expression represses the migration abilities of A549 (*p* < 0.01) and H1650 (*p* < 0.05) cells and knock‐down of NDUFS1 resulted in opposite effects in A549 (*p* < 0.001, *p* < 0.01) and H1650 (*p* < 0.0001, *p* < 0.01) cells (Figure 4C).

To further verify miR‐3130‐5p exerts its function through modulating the activities of NDUFS1, we performed rescue experiments in A549 and H1650 cells. As a result, miR‐3130‐5p overexpression significantly counteracted the inhibition of invasion, migration abilities by NDUFS1 in vitro (*p* < 0.01; Figure 4A–C). In A549 cells, miR‐3130‐5p restored the repressed level of Snail and reduced the increased level of E‐cadherin caused by overexpression of NDUFS1. While in H1650 cells, miR‐3130‐5p regained the expression of Snail which suppressed by overexpression of NDUFS1 (Figure 4D and Figure S6). In summary, our data suggests that miR‐3130‐5p is a strong modulator of NDUFS1 activities in vitro and thereby influences the invasion, migration and EMT of LUAD cells.

### 3.5 miR‐3130‐5p promotes tumor invasion in vivo

**FIGURE 5:**
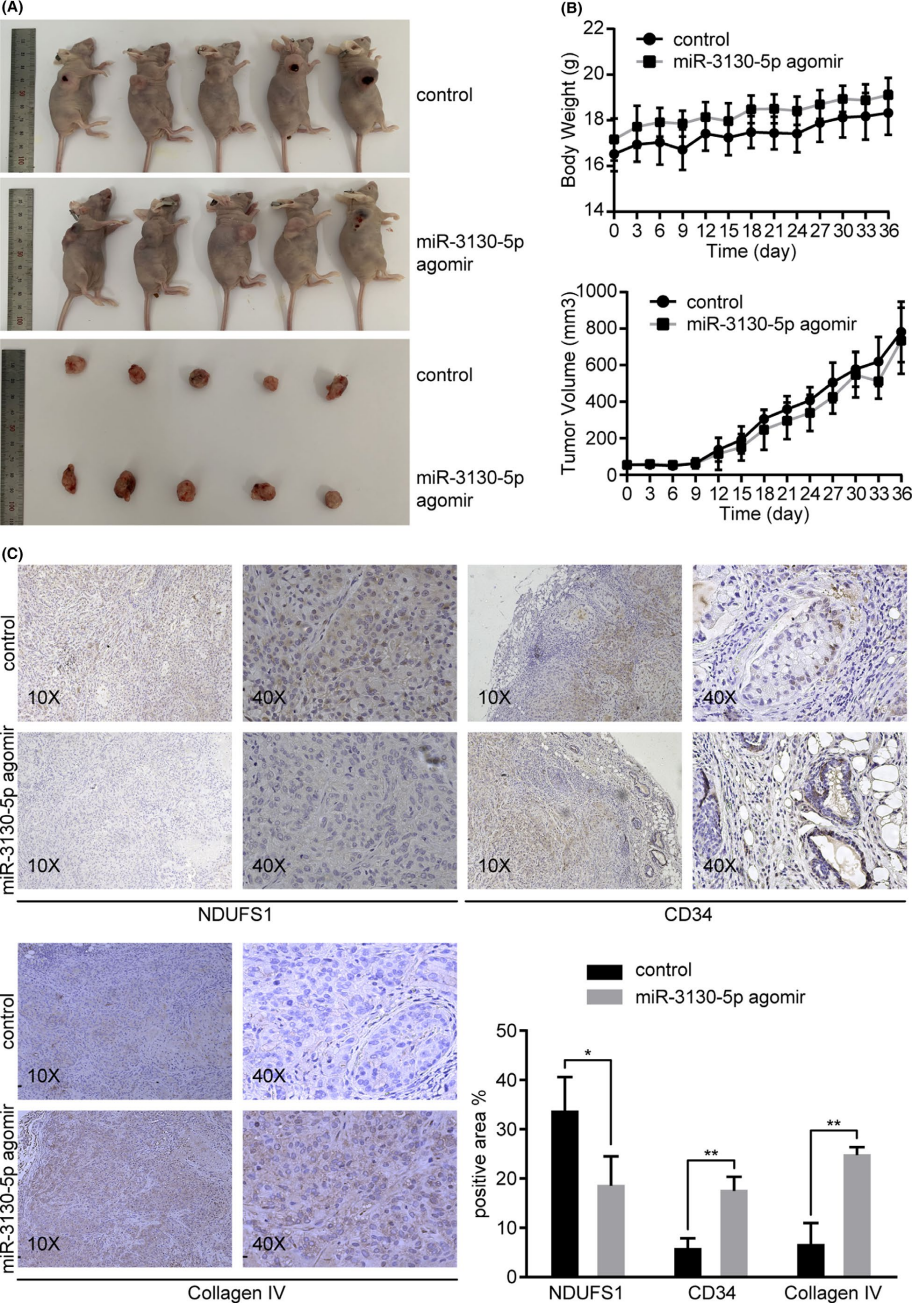
MiR‐3130‐5p promotes tumor invasion in vivo. (A) Mice xenograft models were constructed by injecting A549 cells subcutaneously into nude mice. After the tumors developed to 4 ×4 mm, miR‐3130‐5p agomir and control were injected into mice regularly until mice were sacrificed 4 weeks later. Tumor tissues were resected and examined. (B) There were no significant differences in the body weight and tumor volume between miR‐3130‐5p agomir group and control group. (C) The protein levels of NDUFS1, CD34, and collagen IV were detected by IHC and compared between miR‐3130‐5p agomir group and control. *p < 0.05, **p < 0.01

The in vitro experiments suggest that miR‐3130‐5p functions as a modulator of NDUFS1 with phenotypic impacts in lung cancer. Therefore, we further verified the functions of miR‐3130‐5p in vivo. We established xenograft tumor animal models by subcutaneously injection of A549 cells in BALb/c mice. We intravenously injected miR‐3130‐5p agomir and control to assess the pathological changes. As a result, there were no obvious differences in the tumor volume and body weight of mice between miR‐3130‐5p agomir group and control group (*p* > 0.05; Figure 5A and B), which is consistent with our previous observation in vitro. On the other hand, the immunohistological results showed markedly increased expression of invasion related markers, such as CD34 and collagen IV, in tumors formed in miR‐3130‐5p overexpressing mice compared to the control group (*p* < 0.01; Figure 5C), which suggests that miR‐3130‐5p promotes tumor invasiveness in vivo.

### 3.6 NDUFS1 expression correlates with clinicopathological characteristics of LUAD

We used qRT‐PCR to investigate the expression levels of miR‐3130‐5p and NDUFS1 mRNA in 43 LUAD tissues from patients who received tumor resections. Consistent with previous results, miR‐3130‐5p level was distinguishably up‐regulated (*p* = 5 × 10^−6^; Figure S7) while NDUFS1 mRNA level was down‐regulated in cancerous tissue (*p* = 4.7 × 10^−5^; Figure S7). Moreover, NDUFS1 mRNA level is significantly associated with lymphatic and vascular invasion (OR = 8.67, *p* = 0.004), N stage (OR = 5.50, *p *= 0.019), tumor differentiation (OR = 0.23, *p* = 0.039), tumor maximum diameter (OR = 4.00, *p* = 0.042) (Table 1). Nevertheless, the expression level of miR‐3130‐5p was not significantly related with the clinicopathological characteristics of LUAD (Table S7). Cox regression analysis did not show any risk factors associated with progress‐free survival (PFS) and overall survival (OS) in LUAD patients (Table S8).

**TABLE 1:** Analysis of the correlation between expression level of NDUFS1 in LUAD tissues and clinicopathological characteristics

Clinicopathological characteristics	Total cases	High NDUFS1 level	Low NDUFS1 level	Logit *p*	OR
Age				0.894	1.091
<65	23	12 (60.0%)	11 (57.9%)		
≥65	16	8 (40.0%)	8 (42.1%)		
Sex				0.434	1.671
Female	16	7 (35.0%)	9 (47.4%)		
Male	23	13 (65.0%)	10 (52.6%)		
Stage				0.069	4.121
I+II	28	17 (85.0%)	11 (57.9%)		
III	11	3 (15.0%)	8 (42.1%)		
T stage				0.641	1.429
T1+T2	30	16 (80.0%)	14 (73.7%)		
T3+T4	9	4 (20.0%)	5 (26.3%)		
N stage				0.019*	5.500
N0	24	16 (80.0%)	8 (42.1%)		
N1+N2+N3	15	4 (20.0%)	11 (57.9%)		
Differentiation				0.039*	0.225
Poorly	14	4 (20.0%)	10 (52.6%)		
Well/moderate	25	16 (80.0%)	9 (47.4%)		
ki67				0.152	2.571
<20	19	12 (60.0%)	7 (36.8%)		
≥20	20	8 (40.0%)	12 (63.2%)		
Tumor maximum diameter				0.042*	4.000
<3 cm	21	14 (70.0%)	7 (36.8%)		
≥3 cm	18	6 (30.0%)	12 (63.2%)		
Lymphatic and vascular invasion				0.004**	8.667
Negative	22	16 (80.0%)	6 (31.6%)		
Positive	17	4 (20.0%)	13 (68.4%)		
Nerve invasion				0.261	2.182
Negative	26	15 (75.0%)	11 (57.9%)		
Positive	13	5 (25.0%)	8 (42.1%)		

* *p*<0.05.

** *p*<0.01.

## 4 DISCUSSION

Numerous studies suggest TAL act as eQTL of transcripts with regulatory activities, such as miRNA, lncRNA, or circRNA and thereby influence gene expression in cancer cells. Such regulatory axes are used to explain the biological process underlying non‐coding TAL and to inform new cancer‐related transcripts. In the current study, we identify miR‐3130‐5p as a *cis*‐target of TAL 2q33. Further analysis suggests miR‐3130‐5p is a potential oncogenic microRNA, which intermediates the effects of 2q33 risk locus on NDUFS1 and influence the invasion phenotype of LUAD.

2q33 is reported as a pleiotropic TAL which is associated with nicotine dependence, urinal mercapturic acids concentration in smokers as well as risk of lung cancer and esophageal cancer in Northern Chinese.^30^, ^35^, ^36^ Therefore, the eQTL activity of 2q33 helps understand the pathogenic mechanism of lung cancer. MiR‐3130‐5p is located 70,115 bp from the tag SNP, rs75358501. Prior studies showed that miR‐3130‐5p was associated with diverse diseases.^37^, ^38^, ^39^, ^40^, ^41^, ^42^ It was reported in genomic researches about melanoma and breast cancer.^39^, ^40^ Different expression of miR‐3130‐5p may be involved in breast cancer neo‐adjuvant chemotherapy resistance.^41^ However, the function of miR‐3130‐5p is less known in other cancer types. To our knowledge, the current study is the first report of miR‐3130‐5p as a *cis*‐target of GWAS loci and associated with lung carcinogenesis with a functional role.

Instrumental variable (IV) regression provides a powerful analytical tool for identifying causal relations among germline variants, intermediate transcripts and downstream effectors *in trans*. Many prior studies successfully used IV regression to elucidate the functional impacts of germline variants. Our study uses IV regression to suggest NDUFS1 as an effector *in trans* of 2q33‐miR‐3130‐5p association, which avoids the high false discovery rates (FDR) in direct *trans*‐eQTL analysis and the low statistical power due to limited sample size. The method we described can be used to explain other non‐coding cancer risk variants. In addition, IV regression can also provide a framework to infer causal variants from a set of variants in linkage disequilibrium.

As the downstream effector of 2q33‐miR‐3130‐5p, NDUFS1 is the largest subunit of mitochondria complex I, which plays important role in the process of oxidative phosphorylation and production of ATP. It is well‐known that dysfunction of mitochondria complex I can cause Warburg effects in cancer cells, which means accelerated aerobic glycolysis and cancer progression.^43^, ^44^ NDUSF1 is suggested to exert anti‐cancer effect in ovarian cancer,^45^ clear‐cell renal‐cell carcinoma ^46^ and NSCLC.^34^ Consistent with previous studies, our study demonstrated NDUFS1 acts as a tumor‐suppressor in the invasion, migration and EMT of LUAD cells. Moreover, low expression of NDUFS1 in cancerous tissue is significantly associated with severe lymphatic and vascular invasion pathologically. Thus, NDUFS1 may be a potential biomarker for the clinicopathological diagnosis of LUAD. However, we had not found NDUFS1 level is associated with the OS and PFS of LUAD patients. The possible reason is the heterogeneity in the cohort, that is, patients received different treatments after resection. With limited sample size it is difficult to assess the predictive power of NDUFS1. However, in a larger cohort such as TCGA, we show NDUFS1 expression is significantly associated with clinical outcome of LUAD. Therefore, NDUFS1 is a potential tumor‐suppressor targeted by 2q33‐miR‐3130‐5p axis which influences the invasiveness of LUAD. Our data suggests that NDUFS1 serves as a biomarker for diagnosis and prediction of LUAD.

The current study provides a thorough analyses to confirm the functional impacts of 2q33 and miR‐3130‐5p association through altering NDUFS1 transcription. However, the effect of the regulatory axis needs to be compared with known LUAD biomarkers, such as driver mutations in EGFR, ALK and ROS1.^47^ Although we validated the interaction between miR‐3130‐5p and NDUFS1, there are multiple mechanisms through which miRNA alters the transcript abundances of mRNA. For example, miRNA affects the decay of mRNA, and induces transcriptional gene silencing in mammalian cell (RITS) by directly targeting the promoter. The current analysis does not inform the mechanism through which miR‐3130‐5p inhibit NDUFS1 transcription. In addition, miR‐3130‐5p may interact with other transcripts in LUAD and thereby influences the phenotype of cancer. For example, LncRNA and circRNA competitively bind with miRNA and regulate the expression of downstream target mRNA.^48^, ^49^ Therefore, it is possible that the pathogenicity of miR‐3130‐5p is through other complex pathways in LUAD.

In conclusion, we describe an eQTL analysis in LUAD to reveal the biological mechanism of TAL 2q33. We demonstrate that miR‐3130‐5p is an oncomiRNA which is significantly associated with 2q33 and a modulator of NDUFS1 expression in LUAD. In addition, miR‐3130‐5p suppresses the activity of NDUFS1 and thereby promotes tumor invasiveness and possibly migration and EMT hence a potential biomarker of LUAD. Our analyses identified and verified a novel pathogenic pathway for 2q33 in LUAD through miR‐3130‐5p‐NDUFS1 axis. In addition, we foresee a couple of steps in the future study. First, we can further verify the function of miR‐3130‐5p and NDUFS1 in knockout cell lines and mice. Then, we can establish a large, well‐controlled LUAD cohort to evaluate the clinical significance of 2q33, miR‐3130‐5p, and NDUFS1. We can also verify the invasiveness phenotypes using methods such as in vivo clonogenic assay. These findings will extend our knowledge of lung carcinogenesis and inform the clinical management of LUAD.

## CONFLICT OF INTEREST

All authors declare no conflict of interest.

## ETHICS STATEMENT

The study involving lung cancer patients was approved by the Medical Ethics Council for Researchers of Zhongshan Hospital affiliated to Xiamen University. The ethic approved number is xmzsyyky2017034. All participants were informed and signed the consent forms. The animal experiments were performed according to the principles and procedures formulated by Laboratory Animal Center of Xiamen University.

## Supporting information

Fig S1‐S7

Table S1

Table S2

Table S3

Table S4

Table S5

Table S6

Table S7

Table S8

## Data Availability

All supplementary tables that support the findings of this study are available at https://github.com/xcqxcq/miQTL/tree/main/supplementaryTables online. The data and materials of this study are available from the corresponding author for reasonable requests.
