# Supplementary material for: MiR‐3130‐5p is an intermediate modulator of 2q33 and influences the invasiveness of lung adenocarcinoma by targeting NDUFS1
Source: Cancer Med. 2021 May 12;10(11):3700–14. doi: 10.1002/cam4.3885 (PMC8178510; doi:10.1002/cam4.3885)
Supplement: Supplementary file 1 — Fig S1‐S7 [file CAM4-10-3700-s005.pdf]

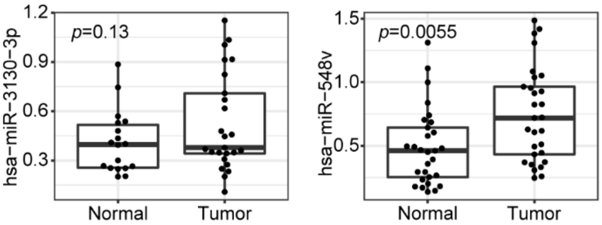

Figure S1. Boxplots of expression of miR-3130-3p and miR-548v in normal lung tissue (Normal）and lung tumor tissue (Tumor) according to TCGA-LUAD database.

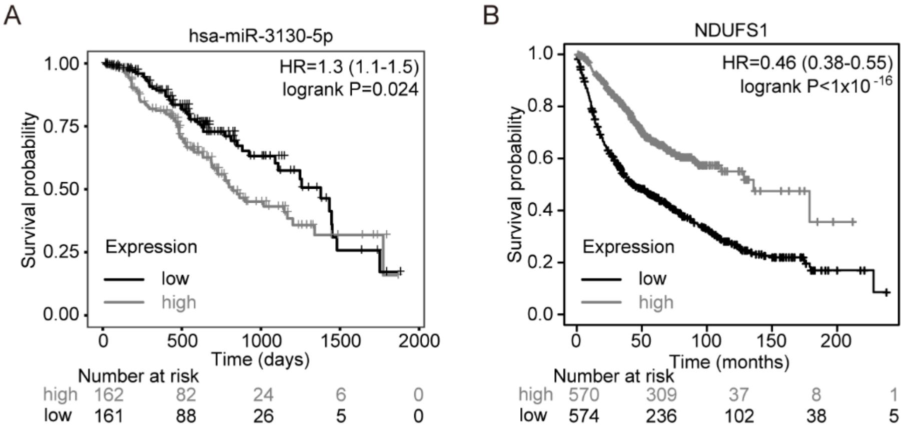

Figure S2. Kaplan-Meier survival plots for miR-3130-5p and NDUFS1 according to stratification of different expression levels in lung cancer tissues.

(A) Kaplan-Meier survival plot for miR-3130-5p in LUAD according to TCGA-LUAD database.

(B) Kaplan-Meier survival plot for NDUFS1 in lung cancer according to KMPLOT-Lung Cancer database.

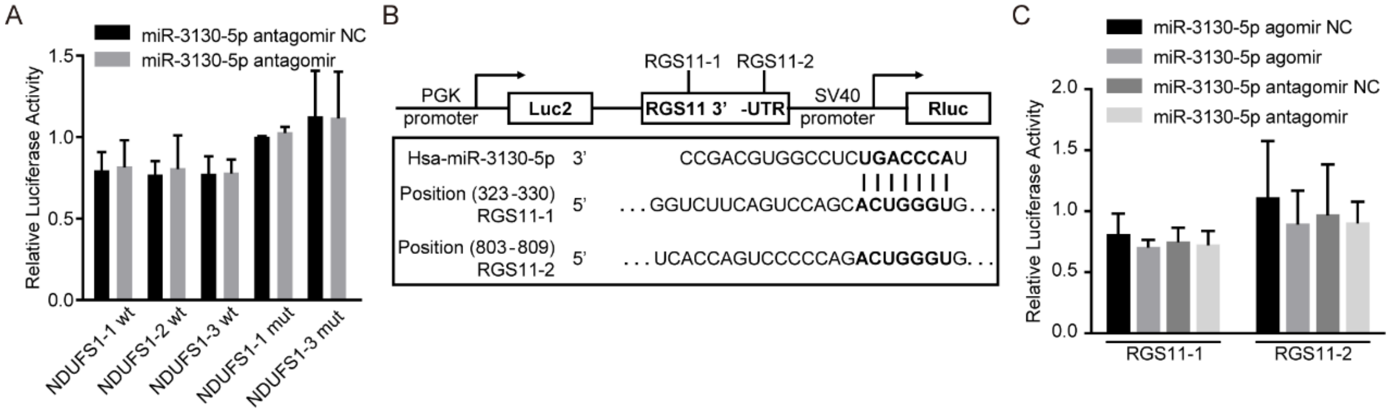

Figure S3. The reporter with NDUFS1 3'UTR showed no response to knock-down of miR-3130-5p and it is demonstrated that RGS11 is not the target gene of miR-3130-5p by luciferase activities assays.

(A) The relative luciferase activities were detected in A549 cells transfected with wild-type or mutant-type NDUFS1 3'UTR and miR-3130-5p antagomir or NC. (B) The schematic shows wild type RGS11 3'UTR (including two different predicted binding sites of miR-3130-5p) was cloned to the pmirGLO Dual-Luciferase miRNA Target Expression Vector. (C) The relative luciferase activities were detected in A549 cells transfected with wild-type RGS11 3'UTR and miR-3130-5p agomir or antagomir and respective controls.

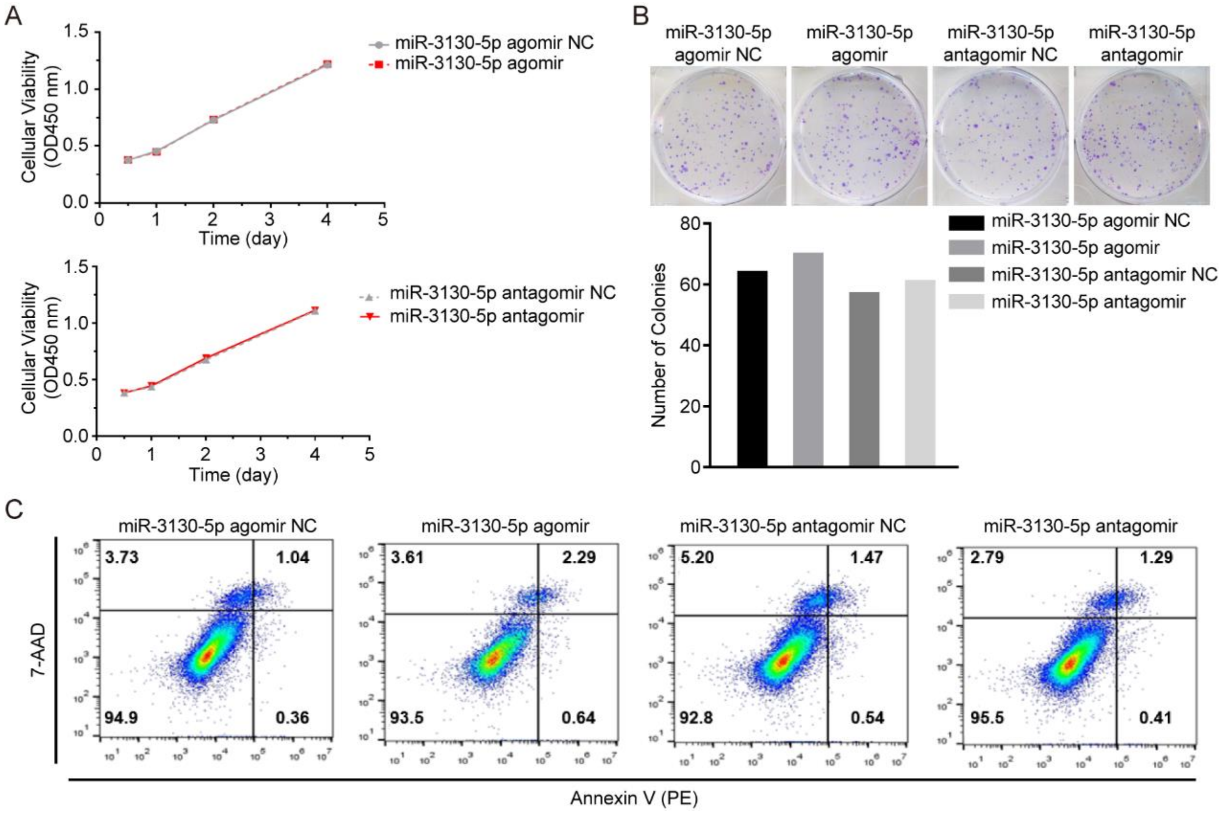

Figure S4. MiR-3130-5p has no effect on the proliferation, colony formation and apoptosis of LUAD cells.

(A) The results of CCK8 assays showed no differences in cellular viabilities occurred when cells were treated with miR-3130-5p agomir or antagomir. (B) The representative images and quantification of crystal violet-stained cell colonies indicated no significant changes among different groups. (C) The apoptosis cells percentages detected by AnnexinV-PI double staining were not significantly different among the indicated groups.

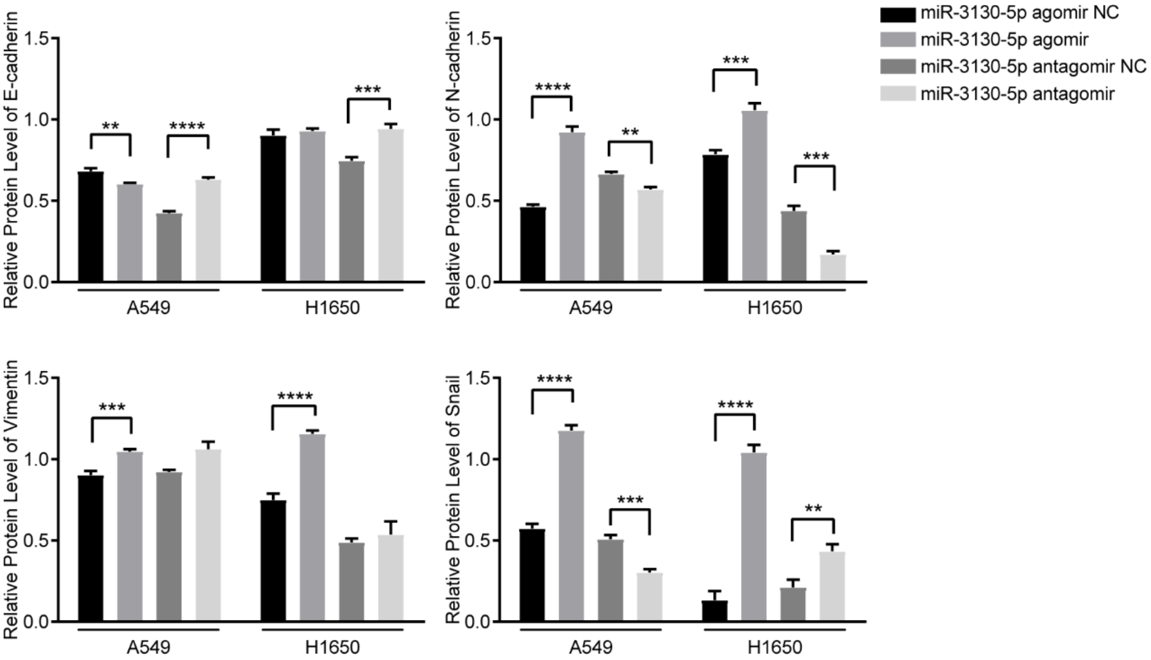

Figure S5. The histograms of EMT related proteins (E-cadherin, N-cadherin, Vimentin and Snail) in A549 and H1650 cells under indicated conditions assayed by western blotting (supplement of Figure 3D). **P<0.01, ***P<0.001, ****P<0.0001.

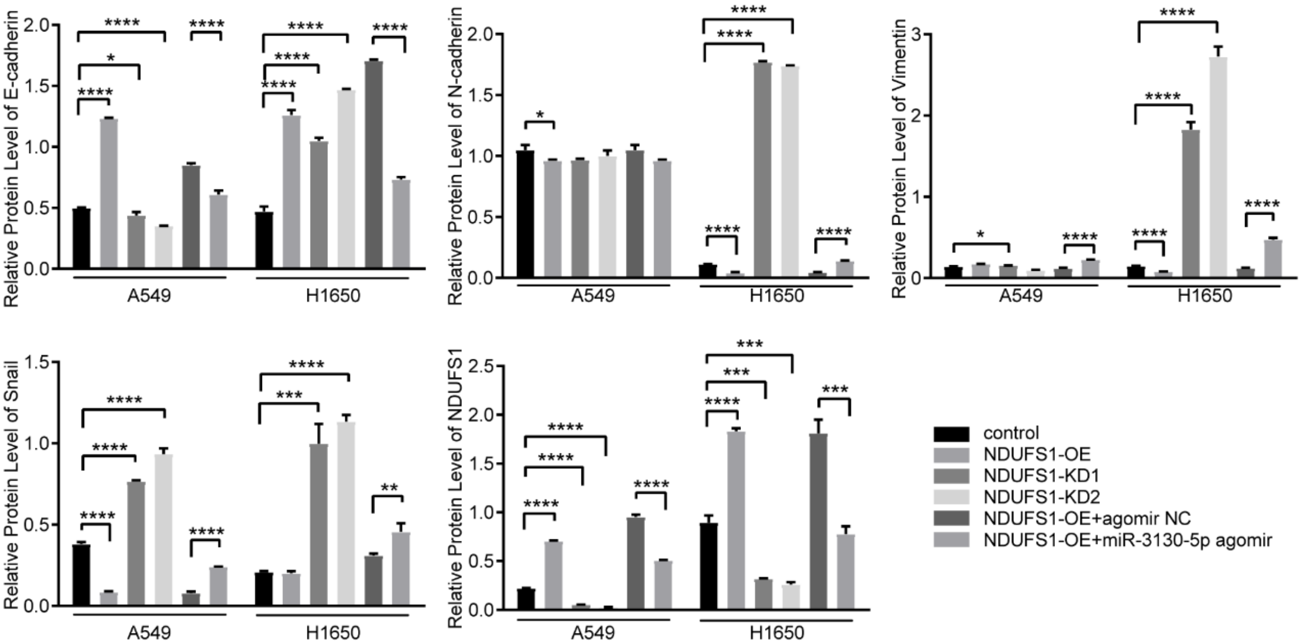

Figure S6. The histograms of EMT related proteins (E-cadherin, N-cadherin, Vimentin and Snail) and NDUFS1 levels in A549 and H1650 cells under indicated conditions assayed by western blotting (supplement of Figure 4D).*P*<0.05, ***P*<0.01, ****P*<0.001, *****P*<0.0001.

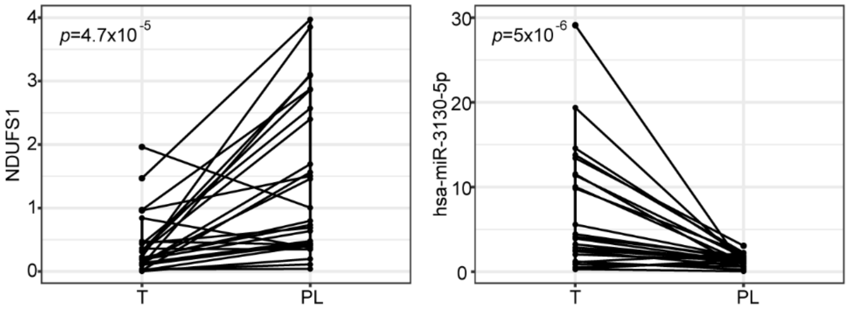

Figure S7. The graph indicates the differences of NDUFS1 and miR-3130-5p expression between LUAD and paracancerous tissues assayed by qRT-PCR. (T represents lung tumor tissue and PL represents paracancerous lung tissue).
